# Clinical outcomes of breast cancer patients treated in phase I clinical trials at University of Colorado Cancer Center

**DOI:** 10.1002/cam4.3487

**Published:** 2020-10-16

**Authors:** Jennifer A. Weiss, Andrew Nicklawsky, Jodi A. Kagihara, Dexiang Gao, Christine Fisher, Anthony Elias, Virginia F. Borges, Peter Kabos, Sarah L. Davis, Stephen Leong, Sue Gail Eckhardt, Jennifer R. Diamond

**Affiliations:** ^1^ University of Colorado School of Medicine Aurora CO USA; ^2^ Division of Medical Oncology University of Colorado Anschutz Medical Campus Aurora CO USA; ^3^ Department of Radiation Oncology University of Colorado Anschutz Medical Campus Aurora CO USA; ^4^ Division of Medical Oncology Dell Medical School University of Texas at Austin Austin TX USA

**Keywords:** immunotherapy, metastatic breast cancer, phase I clinical trials, targeted therapies

## Abstract

Patients with metastatic breast cancer (MBC) refractory to standard of care therapies have a poor prognosis. The purpose of this study was to assess patient characteristics and clinical outcomes for patients with MBC treated on phase I clinical trials. We performed a retrospective review of all patients with MBC who were enrolled in phase I clinical trials at the University of Colorado Cancer Center from January 2012 to June 2018. A total of 208 patients were identified. Patients had a mean age of 57 years and received on average 2.1 (range 0‐10) prior lines of chemotherapy. The majority of patients had hormone receptor‐positive/HER2‐negative breast cancer (58.6%) and 30.3% had triple‐negative breast cancer. The median progression free survival (PFS) was 2.8 months (95% CI, 2.3‐3.9) and median overall survival (OS) was 11.5 months (95% CI, 9.6‐13.2). Independent factors associated with longer PFS in multivariable analysis were treatment in a breast cancer‐selective trial or cohort (*p* = 0.016), age >50 years (*p* = 0.002), and ≤2 prior lines of chemotherapy in the metastatic setting (*p* = 0.025). Phase I clinical trials remain a valuable option for select patients with MBC and enrollment should be encouraged when available.

## Graphical abstract

We performed a retrospective review of all patients with metastatic breast cancer (MBC) who were enrolled in phase I clinical trials at the University of Colorado Cancer Center. Independent factors associated with longer PFS in multivariable analysis were treatment in a breast cancer‐selective trial or cohort, age >50 years, and ≤2 prior lines of chemotherapy in the metastatic setting. Phase I clinical trials remain a valuable option for selecting patients with MBC and enrollment should be encouraged when available.

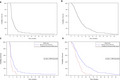

## 1 INTRODUCTION

Breast cancer is the most common cancer in women in the United States and the leading cause of cancer‐related deaths in women worldwide.^1^ Despite the availability of multiple active systemic therapies that can prolong progression‐free survival (PFS) and overall survival (OS), only approximately 27% of patients with metastatic breast cancer (MBC) will be alive at 5 years.^2^ The prognosis and treatment of breast cancer differs by biologic subtype as defined by hormone receptor (HR) expression and human epidermal growth factor receptor 2 (HER2) amplification.^3^, ^4^ The median overall survival for patients with metastatic triple‐negative breast cancer (TNBC) is approximately 12‐18 months, compared to 35‐55 months for HR‐positive/HER2‐negative breast cancer.^5^, ^6^, ^7^, ^8^, ^9^ Despite advances in the treatment of MBC, acquired resistance to therapies is nearly universal.

Phase I clinical trials are a critical step in the development of novel anti‐cancer therapies. Traditionally, phase I clinical trials were designed with limited objectives to characterize the safety profile of new drugs, determine pharmacokinetic and pharmacodynamic properties, and identify the recommended phase 2 dose (RP2D) for future efficacy studies. These trials historically enrolled patients with advanced solid tumors refractory to standard of care therapy, and treatment on these trials resulted in low objective response rates.^10^, ^11^, ^12^ Recently, however, we have witnessed a shift from the development of largely cytotoxic chemotherapeutics to targeted therapies often with a biologic selector incorporated early on in clinical development.

The purpose of this study was to investigate baseline characteristics and clinical outcomes for patients with MBC treated recently in phase I clinical trials at the University of Colorado Cancer Center and to investigate factors associated with clinical benefit, including enrollment into trials or cohorts restricted to breast cancer or a breast cancer subtype.

## 2 MATERIALS AND METHODS

We identified all patients with MBC enrolled in phase I clinical trials at the University of Colorado Cancer Center from 1 January 2012 to 15 June 2018. Phase I oncology clinical trials included all protocols investigating single agent or multi‐agent investigational drugs with phase I or phase Ib in the title. For phase I/II or Ib/II clinical trials, patients enrolled in the phase I portion of the study were included in this analysis. Patients enrolled in a phase II cohort were not included. All investigational treatment was administered at the University of Colorado Cancer Center as part of a clinical trial with institutional review board (IRB) approval and all patients provided written informed consent before enrollment on these trials.

All patient records were reviewed using an electronic medical record system including medical history, laboratory results, and treatment outcomes. Baseline characteristics were collected including: age, date of diagnosis, stage at diagnosis, estrogen receptor (ER) and progesterone receptor (PR) expression, HER2 status, metastatic sites of disease, prior systemic therapies, Eastern Cooperative Oncology Group performance status (ECOG PS), and lab values obtained at the time of enrollment on the phase I clinical trial. ER, PR, and HER2 receptor status was evaluated based on the local pathology report. Other data collected included: reason for study discontinuation (disease progression, drug‐related toxicity, or other), time of treatment discontinuation, disease progression, and death. For patients enrolled sequentially into multiple phase I clinical trials, data was collected for each trial enrollment. This study was performed in accordance with local IRB guidelines and data was stored in a secure online database.

Phase I trials or specific cohorts with enrollment restricted to breast cancer or a particular breast cancer subtype were designated as a breast cancer‐selective trial. Cohorts or trials with enrollment that was limited to MBC and up to one other tumor type were also considered breast cancer‐selective. Phase I trials or cohorts enrolling >2 tumor types were considered traditional phase I trials.

### 2.1 Endpoints and statistical methods

Patient characteristics were summarized using counts and percentages for categorical variables and using the mean and range for continuous variables. The association between patient characteristics and enrollment in breast cancer‐selective vs traditional phase I trials was evaluated with the Mann‐Whitney test for continuous variables, the chi‐square test for categorical variables, and the Fisher exact test for categorical variables with low cell counts. The Mann‐Whitney test was chosen to account for the non‐normal distribution of the continuous variables.

Progression free survival was defined as the time from the initiation of the investigational drug administration to the time of documented disease progression or death. Patients who did not discontinue study treatment due to disease progression were censored at the time of initiation of a new anti‐cancer therapy. PFS and OS analysis was performed using the Kaplan‐Meier method.

Cox proportional hazard regression models were used for univariable associations of patient characteristics with PFS. Candidates for the multivariable model of PFS were identified based on their significance in the univariable analysis and clinical relevance. The predictors for the multivariable Cox model were assessed for linearity amongst continuous variables, interactions with study type, multicollinearity, and proportional hazards. *p*‐values were calculated based on a null hypothesis of no effect against a two‐sided alternative. Analyses were performed using SAS 9.4 (SAS Institute).

## 3 RESULTS

### 3.1 Baseline patient characteristics

A total of 208 patients with MBC were treated in phase I clinical trials at the University of Colorado Cancer Center and included in our analysis. Baseline characteristics are listed in Table 1. The mean age was 57 years (range 31‐80), which included 205 female patients (98.6%) and 3 male patients (1.4%). The majority of patients (58.6%) had HR‐positive/HER2‐negative breast cancer, 30.3% had TNBC, and 11.1% had HER2‐positive breast cancer. ECOG PS was 0 in 38.5% and 1 in 58.5% of patients. The mean number of prior lines of chemotherapy in the metastatic setting was 2.1 (range 0‐10).

**TABLE 1:** Baseline characteristics of patients with metastatic breast cancer treated in phase I trials

	All patients	Breast cancer‐selective	Traditional phase I	*p*‐value
Total	208	167	41	—
Age (years)				
Mean	57	57	55.7	0.52^a^
Range	31‐80	34‐80	31‐72	
Sex				
Male	3 (1.4%)	3 (1.8%)	0 (0%)	1.00^c^
Female	205 (98.6%)	164 (98.2%)	41 (100%)	
Metastatic site				
Bone	142 (68.3%)	118 (70.7%)	24 (58.5%)	0.14^b^
Lung	91 (43.8%)	72 (43.1%)	19 (46.3%)	0.71^b^
Liver	97 (46.6%)	80 (47.9%)	17 (41.5%)	0.46^b^
Brain	28 (13.5%)	25 (15.0%)	3 (7.3%)	0.31^c^
Prior lines of chemotherapy (metastatic setting)				
Mean	2.1	1.8	3.3	<0.0001^a^
Range	0‐10	0‐10	0‐8	
Receptor status				
HR+/HER2−	122 (58.6%)	100 (59.9%)	22 (53.7%)	0.47^b^
HER2+	23 (11.1%)	22 (13.2%)	1 (2.4%)	0.053^c^
TNBC	63 (30.3%)	45 (26.9%)	18 (43.9%)	0.034^b^
ECOG PS				
0	80 (38.5%)	68 (40.7%)	12 (29.3%)	0.26^c^
1	122 (58.6%)	93 (55.7%)	29 (70.7%)	
2	2 (1.0%)	2 (1.2%)	0	
Unknown	4 (1.9%)	4 (2.4%)	0	
Hemoglobin (g/dL)				
Mean	12.5	12.4	12.5	0.74^a^
Albumin (g/dL)				
Mean	3.9	3.9	3.9	0.99^a^
LDH (U/L)				
Mean	294	245	451	0.54^a^

Abbreviations: ECOG PS, Eastern Cooperative Oncology Group Performance Status; HER2, human epidermal growth factor receptor 2; HR, hormone receptor; LDH, Lactate dehydrogenase; TNBC, triple‐negative breast cancer.

*p*‐values were calculated using: ^a^Mann‐Whitney test, ^b^Chi‐square test, or ^c^Fisher's exact test.

Of the total 208 patients, 167 were treated in phase I clinical trials or cohorts considered to be breast cancer‐selective and 41 were enrolled in traditional phase I trials open to patients with many solid tumor types. Baseline characteristics including age, gender, sites of metastatic disease, ECOG PS, and laboratory values did not differ significantly between the two groups (Table 1). However, there were more patients with TNBC treated in traditional phase I trials compared to breast cancer‐selective studies (43.9% vs 26.9%, respectively). Additionally, patients treated in traditional phase I studies had received a higher number of prior lines of chemotherapy in the metastatic setting (mean 3.3, range 0‐8) compared to patients enrolled in breast cancer‐selective trials or cohorts (mean 1.8, range 0‐10).

### 3.2 Phase I clinical trials

The patients were enrolled in 43 phase I clinical trials (Table S1a,b): 26 traditional phase I trials and 17 breast cancer‐selective trials or cohorts; 12 were phase Ib trials. Two of the 17 breast cancer‐selective trials or cohorts enrolled patients with 2 tumor types, including breast and ovarian cancer. The other 15 breast cancer‐selective trials or cohorts had enrollment restricted only to breast cancer or a particular breast cancer subtype. Each breast cancer‐selective trial enrolled a mean of 9.8 patients with MBC per trial (range 1‐22). Each traditional phase I trial enrolled a mean of 1.6 patients with MBC per trial (range 1‐6).

**FIGURE 1:**
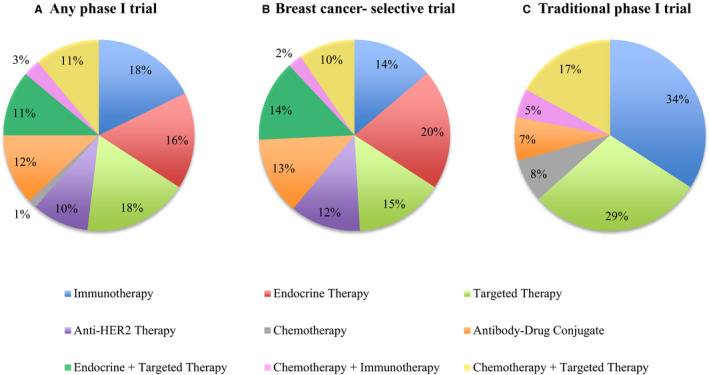
Anti‐cancer therapy class of investigational and standard of care therapies administered to patients with metastatic breast cancer enrolled in (A) any phase I trial, (B) breast cancer‐selective phase I trials, and (C) traditional phase I trials

Patients were treated with investigational and standard of care drugs categorized as: immunotherapy (including immune checkpoint inhibitors and vaccines) (N = 37, 18%), endocrine therapy (estrogen receptor or androgen receptor inhibitors/down‐regulators) (N = 34, 16%), antibody‐drug conjugates (ADCs) (N = 25, 12%), anti‐HER2 agents (N = 20, 10%), other targeted agents (including small molecule inhibitors or monoclonal antibodies) (N = 37, 18%), other targeted agents plus endocrine agents (N = 23, 11%), other targeted agents plus chemotherapy (N = 23, 11%), chemotherapy plus immunotherapy (N = 6, 3%), and chemotherapy alone (N = 3, 1%) (Figure 1). Patients treated in traditional phase I trials received immunotherapy alone or in combination with another agent (39%) more frequently than patients enrolled in breast cancer‐selective trials (16%). Patients enrolled in breast cancer‐selective trials more frequently received endocrine agents and anti‐HER2 agents compared to patients treated in traditional phase I trials (Figure 1).

### 3.3 Clinical outcomes

The majority of patients came off study for disease progression (85%), followed by drug‐related toxicity (7.8%), and other reasons including withdrawal of consent (7.3%). The proportion of patients coming off study for toxicity was similar for breast cancer‐selective (7.3%), and traditional phase I trials (9.8%). One patient was lost to follow‐up and not included in the analysis.

The median PFS for all patients with MBC treated in phase I clinical trials was 2.8 months (95% CI, 2.3‐3.9) and the median OS was 11.5 months (95% CI, 9.6‐13.2) (Table 2). Forty‐three patients were alive at the time of data cutoff (36 in breast cancer‐selective and 7 in traditional phase I trials). Seven patients were censored for OS due to unknown date of death.

**TABLE 2:** Clinical outcomes patients with metastatic breast cancer treated in phase I trials

	Median PFS [mos (95% CI)]	*p* value	Median OS [mos (95% CI)]	*p* value	2‐year survival (95% CI)
Phase I trial type					
All	2.8 (2.3‐3.9)		11.5 (9.6‐13.2)		19.6% (13.9‐26.1)
Breast cancer‐selective	3.7 (2.4‐5.1)	—	12.7 (10.3‐14.8)	—	23.7% (16.8‐31.3)
Traditional phase I	2.1 (1.6‐2.5)	0.0007	8.2 (6.0‐11.1)	<0.0001	3.0% (0.2‐13.3)
Breast cancer subtype					
HR+/HER2−	2.6 (2.1‐3.7)	—	12.1 (10.8‐16.1)		25.1% (16.7‐34.4)
TNBC	2.5 (1.8‐4.2)		10.3 (8.3‐13.6)		8.7% (3.2‐17.7)
HER2+	5.4 (2.1‐6.6)		8.9 (6.6‐33.7)		31.2% (11.0‐54.1)
HR+/HER2−					
Breast cancer‐selective	3.4 (2.2‐5.5)	—	14.8 (11.5‐18.4)	—	—
Traditional phase I	2.1 (1.4‐2.5)	0.0142	7.9 (5.0‐9.6)	<0.0001	
TNBC					
Breast cancer‐selective	2.9 (1.8‐5.1)	—	9.3 (7.9‐14.6)	—	—
Traditional phase I	2.5 (1.5‐4.2)	0.1250	11.1 (6.0‐14.1)	0.19	

Abbreviations: CI, confidence interval; HER2, human epidermal growth factor receptor 2; HR, hormone receptor; mos, Months; OS, overall survival; PFS, progression free survival; TNBC, triple‐negative breast cancer.

**FIGURE 2:**
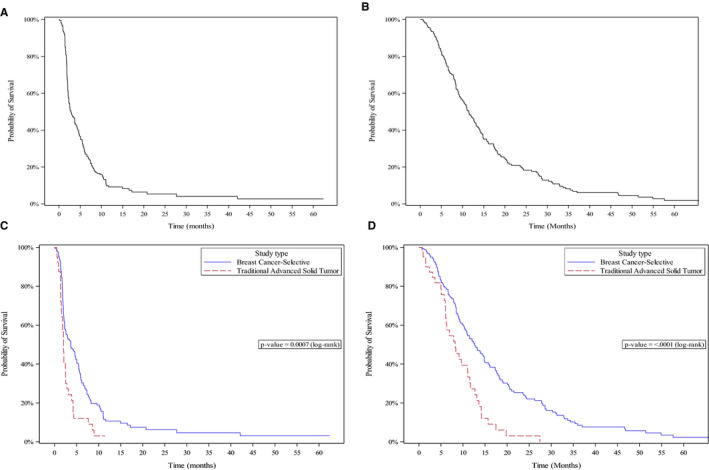
Probability of progression free survival (PFS) and overall survival (OS) using the Kaplan‐Meier method. (A) PFS for all patients with metastatic breast cancer enrolled in phase I trials (median 2.8 months, CI 95%: 2.3‐3.9). (B) OS for all patients (11.5 months, CI 95%: 9.6‐13.2). (C) PFS for patients enrolled on breast cancer‐selective (3.7 months, CI 95%: 2.4‐5.1) and traditional phase I trials (2.1 months, CI 95%: 1.6‐2.5) (*p* = 0.007). (D) OS for patients enrolled on breast cancer‐selective (12.7 months, CI 95%: 10.3‐14.8) and traditional phase I trials (8.2 months, CI 95%: 6.0‐11.1) (*p* < 0.0001)

Patients treated in breast cancer‐selective trials or cohorts had a median PFS of 3.7 months (95% CI, 2.4‐5.1), median OS of 12.7 months (95% CI, 10.3‐14.8), and 2‐year survival of 23.7% (Table 2; Figure 2).

Patients treated in traditional phase I trials had a median PFS of 2.1 months (95% CI, 1.6‐2.5), median OS of 8.2 months (95% CI, 6.0‐11.1), and 2‐year survival of 3.0% (Table 2; Figure 2).

Clinical outcomes by breast cancer subtype are shown in Table 2. The median PFS for patients with HR‐positive/HER2‐negative, TNBC and HER2‐positive MBC were 2.6 months (95% CI, 2.1‐3.7), 2.5 months (95% CI, 1.8‐4.2), and 5.4 months (95% CI, 2.1‐6.6), respectively. OS was not significantly different between these groups.

### 3.4 Exceptional responders to treatment in phase I trials

At the time of our data cutoff, 2 patients remained on study without disease progression. One patient with HR+/HER2− MBC treated with fulvestrant and sapanisertib (Torc1/2 inhibitor, TAK228) remained on study for over 5 years. The other patient had metastatic TNBC and was treated with nab‐paclitaxel and atezolizumab (PD‐L1 inhibitor). She came off study for toxicity, however, has remained without disease progression for over 3.5 years in the absence of any subsequent anti‐cancer therapy.

Fourteen patients (6.7%) enrolled in 7 different trials remained on their phase I study treatment for over 12 months with control of disease (Table S2). This included 8 patients with HR+/HER2− MBC treated with elacestrant (N = 3), fulvestrant +sapanisertib (N = 2), fulvestrant +palbociclib + gadatolisib (N = 2), and enzalutamide (N = 1); 4 patients with TNBC treated with sacituzumab govitecan (N = 2) and nab‐paclitaxel +atezolizumab (N = 2); and 2 patients with HER2‐+ MBC treated with tucatinib +ado‐trastuzumab emtansine. All fourteen patients were in breast cancer‐selective trials.

### 3.5 Independent factors associated with prolonged PFS

By univariate analysis, factors associated with prolonged PFS included: enrollment in a breast cancer‐selective trial or cohort (*p* = 0.001), age >50 years (*p* = 0.003), ≤2 prior lines of chemotherapy in the metastatic setting (*p* = 0.0034), and treatment with a chemotherapy agent in the trial (*p* = 0.044) (Table 3).

**TABLE 3:** Univariate analysis on progression free survival

	Hazard ratio (95% CI)	*p*‐value
Phase I trial type		
Breast cancer‐selective	0.53 (0.36‐0.77)	0.001
Traditional phase I	1.0 (reference)	—
Age (years)		
<50	1.0 (reference)	—
≥50	0.61 (0.44‐0.85)	0.003
Receptor status		
TNBC	1.0 (reference)	—
ER+HER2−	0.84 (0.61‐1.17)	0.30
HER2+	0.71 (0.43‐1.17)	0.18
ECOG PS		
0	1.0 (reference)	—
1‐2	1.11 (0.81‐1.51)	0.52
Prior adjuvant chemotherapy		
Yes	1.08 (0.78‐1.48)	0.65
No	1.0 (reference)	—
Lines chemotherapy metastatic setting		
0‐2	1.0 (reference)	—
≥3	1.61 (1.17‐2.21)	0.003
Brain metastasis		
Yes	0.74 (0.48‐1.13)	0.17
No	1.0 (reference)	—
Liver metastasis		
Yes	1.26 (0.93‐1.70)	0.14
No	1.0 (reference)	—
Lung metastasis		
Yes	0.98 (0.73‐1.32)	0.89
No	1.0 (reference)	—
Albumin (g/dL)		
<3.5	1.0 (reference)	—
≥3.5	0.87 (0.58‐1.30)	0.51
LDH (UI/L)		
<272	1.0 (reference)	—
≥272 (upper limit of normal)	1.01 (0.69‐1.48)	0.95
Phase I trial treatment includes chemotherapy agent		
Yes	0.62 (0.40‐0.99)	0.044
No	1.0 (reference)	—

Abbreviations: CI, confidence interval; ECOG PS, Eastern Cooperative Oncology Group Performance Status; HER2, human epidermal growth factor receptor 2; HR, hormone receptor; LDH, Lactate dehydrogenase; TNBC, triple‐negative breast cancer.

In a COX multivariable analysis, treatment in a breast cancer‐selective trial and age >50 year were associated with a longer PFS and prior treatment with ≥3 prior lines of chemotherapy in the metastatic setting was associated with a shorter PFS (Table 4). TNBC was not an independent predictor of PFS in multivariable analysis.

**TABLE 4:** Multivariable analysis on progression free survival

	Hazard radio (95% CI)	*p*‐value
Phase I trial type		
Breast cancer‐selective	0.61 (0.41‐0.91)	0.016
Traditional phase I	1.0 (reference)	—
Age (years)		
<50	1.0 (reference)	—
≥50	0.60 (0.43‐0.83)	0.002
TNBC		
Yes	1.17 (0.85‐1.61)	0.35
No	1.0 (reference)	—
Lines chemotherapy metastatic setting		
0‐2	1.0 (reference)	—
≥3	1.46 (1.05‐2.04)	0.025

Abbreviations: CI, confidence interval; TNBC, triple negative breast cancer.

## 4 DISCUSSION

The design of phase I clinical trials has evolved over the last decade with a number of phase I clinical trials restricting enrollment to patients with a particular disease type, or even a particular molecular subset of a disease, based on potential or known biomarkers of response.^13^, ^14^ Additionally, anti‐cancer agents undergoing evaluation in phase I clinical trials are increasingly targeted against known oncogenic drivers, immune checkpoints, or other cancer cell targets rather than cytotoxic chemotherapeutics. Breast cancer is well‐positioned to benefit from this shift in drug development given the decades of biomarker‐selected therapies targeting the estrogen receptor and HER2.

While enrollment in phase I clinical trials has historically been viewed by many as a final option for patients who have progressed on all available therapies, these new paradigms in drug development and trial design may provide opportunities for patients to benefit from promising new agents through treatment in phase I clinical trials earlier on in their disease course. While the majority of patients in our study had previously received chemotherapy in the metastatic setting prior to enrolling in a phase I clinical trial, there were many patients who had only received endocrine therapy or were treated with chemotherapy in the first or second line. This trend of earlier enrollment in clinical trials is increasingly being observed in many disease types where targeted agents are being added to standard of care or where a standard of care is not well established.

We hypothesized that in the case of patients with MBC, outcomes for patients treated in phase I clinical trials over the last decade would be generally improved compared to historical case studies. At our center, patients with MBC enrolled in phase I clinical trials had a median PFS of 2.8 months and median OS of 11.5 months. A subset of patients experienced prolonged clinical benefit lasting over 12 months. Treatment with agents in a phase I trial was generally safe with <10% of patients discontinuing therapy for treatment‐related toxicity and no patients died due to treatment‐related toxicity. Age >50 years and prior receipt of ≤2 prior lines of chemotherapy in the metastatic setting were associated with improved clinical outcomes in our dataset, consistent with known prognostic factors in MBC.^15^

Patients treated in breast cancer‐selective trials or cohorts had significantly improved clinical outcomes compared to those treated in traditional phase I trials, however, these groups were heterogeneous in regards to biologic subtype and prior treatment limiting a direct comparison. Clinical outcomes for both groups support consideration of phase I clinical trial enrollment for appropriate patients. Although median overall survival for both groups was <13 months, patients in the breast cancer‐selective trials had a much high 2‐year survival percentage. This is due to the number of exceptional responders within the breast cancer‐selective trials.

Many of our patients who remained on study for over a year with durable clinical benefit were treated with agents that have since gone on to be proven efficacious in phase II or III clinical trials, including tucatinib, sacituzumab govitecan, and atezolizumab with nab‐paclitaxel.^16^, ^17^, ^18^ For example, the small molecule HER2 inhibitor tucatinib was investigated in combination with capecitabine or ado‐trastuzumab emtansine in a phase Ib study with enrollment restricted to HER2+ breast cancer designed to evaluate the safety and tolerability of the combination and assess preliminary efficacy. Tucatinib has since been granted breakthrough therapy designation by the Food and Drug Administration (FDA) and the HER2CLIMB trial recently demonstrated an improvement in PFS and OS with the addition of tucatinib to capecitabine and trastuzumab in patients with previously treated HER2+ MBC.^16^

Patients with MBC have historically been underrepresented in phase I clinical trials despite often having better clinical outcomes compared to other tumor types.^19^ This may be due to the large armamentarium of efficacious treatment options for patients with MBC compared to other cancer types, which can lead to later referrals to a phase I unit when patients may no longer be appropriate for clinical trial enrollment due to a decline in performance status or impaired organ function. There may also be apprehension by patients and providers to enroll in a phase I clinical trial due to the unknown toxicity and efficacy of new investigational agents.^20^ Recent trends in rational drug development and patient selection in phase I trials may result in changes in these patterns.

## 5 CONCLUSION

Phase I clinical trials remain a valuable option for select patients with MBC and enrollment should be encouraged when available. Recent advances in targeted therapies and personalized medicine have likely contributed to improved outcomes observed for patients with MBC treated in phase I clinical trials.

## CONFLICT OF INTEREST

The authors have no relevant conflicts of interest.

## PROTECTION OF HUMAN SUBJECTS

This study was conducted in accordance with the Declaration of Helsinki and local guidelines. The project was conducted following approval by an institutional review board and with a waiver of consent given the retrospective nature of this study and the lack of collection of identifying information.

## Supporting information


**Table S1**

## Data Availability

The complete dataset used in this study is not publicly available.
