# Supplementary material for: Clinical outcomes of breast cancer patients treated in phase I clinical trials at University of Colorado Cancer Center
Source: Cancer Med. 2020 Oct 16;9(23):8801–8. doi: 10.1002/cam4.3487 (PMC7724484; doi:10.1002/cam4.3487)
Supplement: Supplementary file 1 — Table S1 [file CAM4-9-8801-s001.docx]

**Supplemental Table 1a: Breast Cancer-Selective Phase I Trials**

| **Trial** | **Patients (N)** | **Treatment class** | **Drugs administered** |
| --- | --- | --- | --- |
| NCT01597193 | 22 | endocrine | enzalutamide |
| NCT02003092 | 6 | targeted | RX-5902 |
| NCT01983501 | 13 | anti-HER2 | tucatinib,+ trastuzumab emtansine |
| NCT02049957 | 16 | endocrine + targeted | fulvestrant/exemestane + sapanisertib |
| NCT01226316 | 8 | targeted | capivasertib |
| NCT02338349 | 12 | endocrine | elacestrant |
| NCT02386501 | 1 | anti-HER2 | ADXS31-164 |
| NCT02684032 | 3 | endocrine + targeted | fulvestrant + palbociclib + gedatolisib |
| NCT02892123 | 2 | anti-HER2 | ZW25 |
| NCT01082068 | 4 | endocrine + targeted | XL147 + letrozole |
| NCT01556789 | 16 | immunotherapy | ONT-10 |
| NCT01633970 | 4 | chemotherapy + immunotherapy | atezolizumab + nab-paclitaxel |
| NCT01631552 | 22 | antibody-drug conjugate | sacituzumab govitecan |
| NCT03054363 | 4 | anti-HER2 | tucatinib + letrozole + palbociclib |
| NCT01973309 | 16 | chemotherapy + targeted | paclitaxel + vantictumab |
| NCT02270372 | 7 | immunotherapy | ONT-10 + varlilumab |
| NCT02719691 | 11 | targeted | sapanisertib + alisertib |

**Supplemental Table 1b: Traditional Solid Tumor Phase I Trials**

| **Trial** | **Patients (N)** | **Treatment class** | **Drug administered** |
| --- | --- | --- | --- |
| NCT00585195 | 1 | targeted | crizotinib |
| NCT00878189 | 1 | targeted | PF-03084014 |
| NCT01004224 | 2 | targeted | infigratinib |
| NCT01347866 | 2 | targeted | PF-04691502, PD-0325901 |
| NCT01992341 | 1 | chemotherapy + targeted | paclitaxel + trebananib |
| NCT01633970 | 2 | chemotherapy + immunotherapy | atezolizumab + bevacizumab +/- FOLFOX |
| NCT01778439 | 2 | targeted | brontictuzumab |
| NCT01967043 | 1 | chemotherapy | oral paclitaxel + HM30181AK |
| NCT02099058 | 1 | antibody-drug conjugate | telisotuzumab vedotin |
| NCT02122146 | 2 | antibody-drug conjugate | PF-06664178 |
| NCT02250157 | 2 | chemotherapy | oral irinotecan + HM30181AK |
| NCT02219724 | 1 | immunotherapy | MOXR0916 |
| NCT02298387 | 1 | targeted | navicixizumab |
| NCT02366949 | 3 | chemotherapy + targeted | paclitaxel + BAY1217389 |
| NCT02482441 | 1 | targeted | rosmantuzumab |
| NCT02471846 | 1 | immunotherapy | atezolizumab + navoximod |
| NCT02410512 | 2 | immunotherapy | atezolizumab + MOXR0916 |
| NCT02561234 | 1 | targeted | pegzilarginase |
| NCT02635672 | 1 | targeted | BAY1251152 |
| NCT02134067 | 1 | chemotherapy + targeted | paclitaxel + TAS-119 |
| NCT02731742 | 2 | immunotherapy | MK-1966 + SD-101 |
| NCT01827384 | 1 | chemotherapy + targeted | carboplatin + adavosertib |
| NCT03098550 | 1 | immunotherapy | daratumumab + nivolumab |
| NCT02964013 | 6 | immunotherapy | pembrolizumab + vibostolimab |
| NCT02737475 | 1 | immunotherapy | nivolumab + BMS-986178 |
| NCT01920061 | 1 | chemotherapy + targeted | docetaxel + gedatolisib |

Abbreviations: NCT, national clinical trial; N, number

**Supplemental Table 2: Patients with exceptional response (PFS > 1 year)**

| Patient (n=14) | PFS (days) | Subtype | Trial type | Treatment class | Drug administered |
| --- | --- | --- | --- | --- | --- |
| 1 | 455 | HR+/HER2- | BC- selective | Endocrine | Enzalutamide |
| 2 | 632 | HER2+ | BC- selective | Anti-HER2 | Tucatinib + trastuzumab emtansine |
| 3 | 1281 | HER2+ | BC- selective | Anti-HER2 | Tucatinib + trastuzumab emtansine |
| 4 | 1898 | HR+/HER2- | BC- selective | Endocrine + targeted | Fulvestrant + sapanisertib |
| 5 | 843 | HR+/HER2- | BC- selective | Endocrine + targeted | Fulvestrant + sapanisertib |
| 6 | 399 | HR+/HER2- | BC- selective | Endocrine | Elacestrant |
| 7 | 339 | HR+/HER2- | BC- selective | Endocrine | Elacestrant |
| 8 | 551 | HR+/HER2- | BC- selective | Endocrine | Elacestrant |
| 9 | 398 | HR+/HER2- | BC- selective | Endocrine + targeted | Fulvestrant + palbociclib + gedatolisib |
| 10 | 386 | HR+/HER2- | BC- selective | Endocrine + targeted | Fulvestrant + palbociclib + gedatolisib |
| 11 | 637 | TNBC | BC- selective | Chemotherapy + immunotherapy | Abraxane + atezolizumab |
| 12 | 1455 | TNBC | BC- selective | Chemotherapy + immunotherapy | Abraxane + atezolizumab |
| 13 | 503 | TNBC | BC- selective | Antibody-drug conjugate | Sacituzumab govitecan |
| 14 | 523 | TNBC | BC- selective | Antibody-drug conjugate | Sacituzumab govitecan |

Abbreviations: PFS, progression free survival; HR, hormone receptor; HER2, human epidermal growth factor receptor 2; TNBC, triple-negative breast cancer; BC, breast cancer
